# Neutrophils from renal cell carcinoma patients show increased expression of immunosuppressive and angiogenic gene profile

**DOI:** 10.1186/2051-1426-1-S1-P192

**Published:** 2013-11-07

**Authors:** Dana R Obery, Patricia A Rayman, Yu Yang, Jennifer S Ko, James H Finke

**Affiliations:** 1 Immunology, Learner Research Institute at the Cleveland Clinic, Cleveland, OH, USA

## 

Myeloid derived suppressor cells (MDSC) are detected at elevated levels in the peripheral blood mononuclear cells (PBMC) of human renal cell carcinoma (RCC) patients and have been shown to mediate tumor growth by promoting angiogenesis and immune suppression (Zea et al., 2005, Ko et al., 2009). Granulocytic-MDSCs (G-MDSCs), the dominant population in RCC patients, have been shown to suppress T-cell function in RCC patients (Rodriguez et al., 2009; Schmeilau and Finn, 2001; Zea et al., 2005). However, less is known regarding the role of neutrophils in promoting tumor growth in RCC patients and their relationship to G-MDSC. In this study we compared the suppressive and angiogenic profile of neutrophils from RCC patients to those of normal donors and the impact that RCC conditioned media has in altering neutrophils normal function. We showed that IFNγ production of T-cells stimulated with anti-CD3/antiCD28 antibodies (n=4) was suppressed by neutrophils pretreated with SK-RC-26b tumor supernatant (TCM) when compared to neutrophils not exposed to TCM. Further experiments showed that neutrophils from RCC patients had increased expression compared to normal healthy donor neutrophils of genes Vegfa (15.28 fold increase), Vegfb (10.84 fold increase), Tgfb1 (3.31 fold increase), IL10 (23.45 fold increase), IDO1 (4.60 fold increase), Nox1 (9.33 fold increase), and Prokr2 (4.14 fold increase) utilizing custom pro-angiogenic and immunosuppressive RT-PCR arrays (n=4). Furthermore, human angiogenesis Proteome Profile Arrays (n=2) were performed comparing RCC patient and normal healthy donor neutrophils and exhibited an increased level of Activin A (94.5% increase), Angiogenin (70% increase), Angiostatin/ plasminogen (56% increase), IGFBP-1 (188% increase), IGFBP-2 (263% increase),TGF-B1 (157.5% increase), Pentraxin 3 (140% increase), PD-ECGF (184.5% increase), Serpin E1 (196% increase), and Serpin F1 (114.5% increase) in RCC patient neutrophils. Normal neutrophils cultured with ACHN tumor supernatant for 18 hours (n=2) had up-regulation of proteins CXCL16 (111% increase), FDF-7 (82% increase), Serpin E1 (470.5% increase), and uPA (198.5% increase) when compared to normal healthy donor’s neutrophils cultured 18 hours in media. In summary, we observed up-regulation of several pro-angiogenic/ immunosuppressive genes in RCC patient neutrophils, suggesting RCC patient neutrophils may promote tumor escape. In addition, the normal healthy donor neutrophils cultured with ACHN tumor supernatant produces several angiogenic proteins suggesting the tumor microenvironment could be providing factors to mediate these angiogenic functions in RCC patient’s neutrophils.

